# Comprehensive insights into the roles of eosinophils in the mucosal immunity of gastrointestinal tract: defenders, destructors, and modulators

**DOI:** 10.3389/fimmu.2026.1856746

**Published:** 2026-06-05

**Authors:** Xueyan Chen, Kaiwen Li, Xin Jin, Xuxia He, Zhirong Du, Shuang Liu, Shijuan Jiang, Chengzhu Ou, Jingnan Li, Ji Li

**Affiliations:** 1 Department of Gastroenterology, Peking Union Medical College Hospital, Chinese Academy of Medical Sciences & Peking Union Medical College, Beijing, China; 2 Department of Allergy, Peking Union Medical College Hospital, Chinese Academy of Medical Sciences & Peking Union Medical College, Beijing, China; 3 Key Laboratory of Gut Microbiota Translational Medicine Research, Chinese Academy of Medical Sciences, Beijing, China

**Keywords:** cross-tissue modulation, functional heterogeneity, gastrointestinal eosinophils, gastrointestinal homeostasis maintenance, immune modulation, mucosal immunity

## Abstract

Eosinophils have long been recognized as terminal effector cells in type 2 immunity; however, emerging evidence positions them as central regulators of intestinal homeostasis, immune modulation, and tissue adaptation. This review provides a comprehensive overview of gastrointestinal eosinophils, integrating recent advances in their development, regulatory mechanisms, functional subsets, and pleiotropic roles across tissues. The developmental and homing pathways that guide eosinophils to the gut are first discussed, with an emphasis on niche-derived signals that control their survival and localization. The functional heterogeneity of intestinal eosinophils is then highlighted, revealing marked transcriptional and spatial diversification. The diverse functions of gut eosinophils are further summarized, including their contributions to pathogen defense, tissue repair, mutual regulation with the microbiota, anti-tumor, and interactions with immune cells. Finally, the cross-tissue orchestration of eosinophil behavior is examined, with a focus on the gut–immune axis. By synthesizing current knowledge, this review aims to present a holistic picture of gastrointestinal eosinophil biology and to inspire new perspectives for understanding and treating eosinophil-associated intestinal disorders.

## 1 Introduction

Eosinophils were first identified by Paul Ehrlich in the 19th century, and named for their eosinophilic granules, which stain bright red with the acidic dye eosin (1). Traditionally, eosinophils have been regarded primarily as effector cells in antiparasitic immunity and allergic responses, mediating tissue damage and inflammation (2). However, this viewpoint has overlooked their significant roles in physiological homeostasis, particularly within the mucosal immune system.

The gastrointestinal tract, the body’s largest mucosal immune organ, harbors a substantial population of eosinophils in its lamina propria under homeostatic conditions. These cells are not merely destructive inflammatory cells, and play critical roles in maintaining gut homeostasis through immune regulation, barrier maintenance, and tissue repair (3). Recent advances in technologies such as single-cell sequencing and spatial transcriptomics have deepened our understanding of eosinophils, revealing their heterogeneity and functional diversity, especially in the mucosal immunity of the gastrointestinal tract (4).

This review aims to systematically summarize the diverse immune functions of gastrointestinal eosinophils in both homeostatic and pathological states, focusing on their interactions with other immune cells and cross-tissue regulatory networks. We strive to construct a comprehensive framework for the functions of gastrointestinal eosinophils, providing a theoretical basis for exploring underlying mechanisms.

## 2 The development and regulatory mechanism of eosinophils

### 2.1 Development and differentiation of eosinophils

Eosinophils, originating from hematopoietic stem cells (HSCs) in the bone marrow, follow a tightly regulated development pathway. HSCs first differentiate into common myeloid progenitors (CMPs). Subsequently, under the regulation of transcription factors such as GATA-1, the C/EBP family, PU.1, and interferon consensus sequence binding protein (ICSBP) (5–7), CMPs differentiate into eosinophil-committed progenitors (EoPs), which co-express CD34 and interleukin-5 receptor alpha (IL-5Rα) (8, 9). Lineage-specific markers such as Siglec-8 and chemokine C-C motif receptor (CCR3) gradually emerge as differentiation progresses (10).

Mature eosinophils initially enter the circulatory system from the bone marrow and then migrate to specific tissues under the guidance of chemokines and adhesion molecules. Eotaxin, a member of the Chemokine C-C motif ligand (CCL) family, includes eotaxin-1/CCL11, eotaxin-2/CCL24, and eotaxin-3/CCL26. As an eosinophil-selective chemokine, eotaxin induces eosinophil chemotaxis by binding to the CCR3 on the surface of eosinophils (11, 12). IL-5 can further enhance eotaxin-induced eosinophil recruitment (13). In the steady state, Matthews et al. demonstrated that eotaxin in tissues such as the jejunum and thymus also plays an irreplaceable regulatory role in maintaining the basal level of eosinophils (14). In the inflammatory state, eotaxin is expressed in various epithelial and endothelial cells, as well as in eosinophils themselves, mediating the activation and recruitment of eosinophils.

In addition, other members of the CCL family, such as RANTES (CCL5), are also implicated in eosinophil regulation. RANTES can activate eosinophil oxidative metabolism, promote their degranulation into a low-density activated state (15, 16), which contributes to the pathogenesis of asthma and food allergies. Conversely, eosinophils can also regulate RANTES expression by signaling to epithelial cells. Tereda et al. demonstrated that eosinophils adhesion to human nasal epithelial cells (HNECs) inhibits RANTES production in these cells (17). Monocyte chemoattractant protein-1 (MCP-1/CCL2) binds to chemokine receptors (e.g., CCR3) on the surface of eosinophils, thereby inducing their recruitment to the site of inflammation (18–20). Macrophage inflammatory protein 1α (MIP-1α/CCL3) activates eosinophils through specific receptors and promotes their degranulation (21).

Adhesion molecules are indispensable for eosinophil tissue homing. Integrins, as transmembrane receptors, facilitate the adhesion and rolling to the vascular endothelium, and subsequent migration into target tissues. Eosinophils express seven integrin heterodimers, including α4β1 (VLA-4), αMβ2 (Mac-1), αDβ2, α4β7, α6β1, αLβ2 (LFA-1), and αXβ2. These heterodimers bind to adhesion molecules on the endothelial cell surface, such as vascular cell adhesion molecule 1 (VCAM-1), MAdCAM-1, and ICAM-1, and are activated by cytokines like IL-5 and granulocyte-macrophage colony stimulating factor (GM-CSF), thereby promoting eosinophil trans-endothelial migration (22). Vedolizumab, a monoclonal antibody targeting α4β7 integrin, reduces the hormonal burden and improves histological manifestations in certain patients with eosinophilic gastroenteritis (EoGE) (23). Selectins are integral to eosinophil adhesion, with P-selectin binding to P-selectin glycoprotein ligand-1 (PSGL-1) on eosinophils to mediate rolling adhesion to endothelial cells, and E-selectin participating in the late rolling and stable attachment of eosinophils (24, 25).

### 2.2 Regulation of eosinophils by cytokines

The survival, differentiation, maturation, and migration of eosinophils are highly dependent on specific cytokines, with IL-5 playing a core regulatory role in promoting EoPs expansion in both homeostatic and inflammatory states (26). Anti-IL-5 therapies, such as mepolizumab, can inhibit EoPs amplification, thereby reducing peripheral blood eosinophil counts, while differentiated mature eosinophils retain their function (27). In synergy with IL-5, IL-3 and GM-CSF can also promote the survival, proliferation, and differentiation of bone marrow EoPs (28). Previously, it was believed that eosinophils matured in the bone marrow before entering the bloodstream and tissue colonization. However, recent studies have shown that CD34^+^IL-5Rα^+^ EoPs can be found in the bronchial mucosa and differentiate into mature eosinophils in response to local IL-5 signaling within the inflammatory microenvironment (29). IL-4 and IL-13 are classic Th2 cytokines secreted by Th2 cells, eosinophils, mast cells (MCs), Type 2 innate lymphoid cells (ILC2), and others. They activate the downstream STAT6 pathway via co-receptors. Odiase et al. demonstrated that in eosinophilic esophagitis (EoE), STAT6 induces calcium release and influx from the endoplasmic reticulum, which in turn promotes eotaxin-3 secretion, thereby activating eosinophils (30). Meanwhile, IL-4 and IL-13 are also capable of upregulating VCAM-1 on the vascular endothelium, enhancing eosinophil adhesion and transendothelial migration.

In inflammatory states, epithelial cells exposed to external allergens or pathogens can release alarmins such as thymic stromal lymphopoietin (TSLP) and IL-33. TSLP exists in two subtypes: long and short. Short TSLP, predominantly expressed in the steady state, resists pathogens and exhibits anti-inflammatory properties. In contrast, long TSLP activates MCs, dendritic cells (DCs), and T cells via binding to TSLP receptors, thereby exerting pro-inflammatory effects (31). Shoda et al. reported significant elevations in serum TSLP and IL-33 levels in infants with EoGE (32). Guo et al. demonstrated, through reverse transcription-polymerase chain reaction (RT-PCR), that short TSLP mRNA was reduced in EoGE patients, while long TSLP mRNA was significantly increased and positively correlated with mucosal eosinophil peak count (33). IL-33 has also been identified as a key regulator of eosinophil differentiation and function in intestinal inflammation, synergistically activating the downstream NF-κB/MAPK signaling pathway with interferon-γ (IFN-γ) to maintain eosinophil activation (34).

In addition to the classic cytokines mentioned above, several novel regulatory factors have been identified in recent years as research has advanced. IL-18, a member of the IL-1 family, primarily induces IFN-γ production and promotes type 1 inflammatory responses. Venkateshaiah et al. demonstrated that eosinophils from tissue samples of asthma and EoE patients, unlike those from healthy individuals, express both CD101 and CD274 (35). IL-18 can convert naïve eosinophils into this pathogenic subtype *in vitro* (35). Verma et al. used the *Fabpi* promoter to specifically overexpress IL-18 in intestinal epithelial cells and observed significant eosinophil infiltration in the intestines of these mice, which also displayed the CD101^+^CD274^+^ phenotype, along with elevated expression of major basic protein (MBP) and eosinophil peroxidase (EPO) (36). Furthermore, IL-18 overexpression in the intestines of IL-5-deficient and IL-13-deficient mice still induced significantly higher eosinophil infiltration compared to wild-type mice, suggesting that the regulatory mechanism on eosinophils induced by IL-18 may be independent of classical pathways such as IL-5 and IL-4/IL-13 (36). Signal regulatory protein alpha (SIRPα/CD172a) and P2Y14 were found to play an important role in inhibiting eosinophil apoptosis. Garcia et al. demonstrated that the small intestine constitutively expresses high levels of the inhibitory receptor SIRPα on eosinophils, which inhibits eosinophil degranulation and apoptosis, thereby maintaining intestinal eosinophil homeostasis (37). In mice with ligand CD47 deficiency, increased activation and reduced numbers of intestinal eosinophil degranulation markers were observed. P2Y14, a purinergic receptor belonging to the P2Y receptor family, is expressed in various immune cells and regulates inflammation through binding to ligands such as uridine diphosphate-glucose (UDPG). Ma et al. found that the UDPG-P2Y14 pathway regulates macrophage inflammatory activation via downstream STAT1 signaling (38). In ulcerative colitis (UC), Liu et al. reported that UDPG activates ERK1/2 signaling through the P2Y14 receptor on eosinophils, inhibits eosinophil apoptosis, and enhances the expression of inflammation-related genes, thereby driving intestinal inflammation (39).

## 3 Functional subtypes of eosinophils

Eosinophils are extensively distributed in the gastrointestinal tract, primarily within the lamina propria of the gastrointestinal wall, and constitute a vital component of the gastrointestinal mucosal immune system. However, gastrointestinal eosinophils are not a homogeneous population. They can differentiate into distinct functional subtypes under varying conditions to perform diverse functions, such as pro-inflammatory and anti-inflammatory actions, causing tissue damage or promoting tissue repair (**Tables 1**, **2**). The advent of single-cell omics and other advanced technologies has provided robust support for elucidating the functional subsets of eosinophils with distinct molecular markers and regulatory patterns (4).

**Table 1:** Summary of molecular markers, distribution, and functions of eosinophil functional subtypes.

Subtype	Species	Tissue/distribution	Key molecular markers	Main function	Reference
Lamina propria-associated subtype	Mouse	Gastrointestinal lamina propria	CD11c^-/lo^, high SSC, low Siglec-F/CD11b	Resting state	(40)
Intraepithelial-associated subtype	Mouse	Gastrointestinal epithelium	CD11c^hi^, decreased SSC, increased Siglec-F/CD11b	Activated state	
A-Eos	Mouse	Gastrointestinal tract (enriched)	High expression of IL-1β, TNF, S100a family, CD80, PD-L1	Antimicrobial, immune regulation	(34)
B-Eos	Mouse	GI tract, spleen, blood, bone marrow	High expression of MMP-9, TGF-β1	Tissue remodeling	
Pro-inflammatory subtype	Human	Esophagus (EoE patients)	EpCAM^+^, CD44^+^	Produces CCL26, TNF-α; disrupts epithelial barrier	(42)
Pro-fibrotic subtype	Human	Esophagus (EoE patients)	CD69^+^, IL-1R1^+^	Secretes TGF-β1, VEGF-A; promotes collagen deposition and fibrosis/stricture	
rEos	Mouse	Lung parenchyma	Ring-shaped nucleus, Siglec-F^int^, CD62L^+^, CD101^low^	High expression of regulatory genes (Anxa1, Runx3, Ldlr)	(43)
iEos	Mouse	Peribronchial area, bronchoalveolar lavage fluid	Lobulated nucleus, Siglec-F^hi^, CD62L^-^, CD101^hi^	High expression of pro-inflammatory genes (Tlr4, C3ar1, Il6)	
SIE1	Mouse	Small intestine	Transcriptomic signature	Metabolic regulation	(47)
SIE2	Mouse	Small intestine	Transcriptomic signature	Mucosal immunity	
SIE3	Mouse	Small intestine	Transcriptomic signature	Stress/inflammation	
Sp-Eos	Mouse	Small intestinal villi	CD22^+^	Long-lived, spatial specialization	
M-Eos	Mouse	Small intestinal crypts	CD11a^+^	Short-lived, spatial specialization	

**Table 2:** Comparison of human eosinophil functions and phenotypes across different tissues and disease states.

Tissue/site	Disease/state	Main functional tendency	Phenotypic characteristics	Key effector molecules	Reference
Colon	IBD	Activated, immunomodulatory	Colocalization with CD4^+^ T cells; similar to mouse A-Eos	CD80, PD-L1	(34)
Esophagus	EoE – pro-inflammatory	Pro-inflammatory, epithelial barrier disruption	EpCAM^+^, CD44^+^	CCL26, TNF-α	(42)
Esophagus	EoE – pro-fibrotic	Pro-fibrotic, stricture formation	CD69^+^, IL-1R1^+^	TGF-β1, VEGF-A	
Lung	Healthy/non-asthmatic	Resident, regulatory	Siglec-8^+^, CD62L^+^, IL-3R^low^	—	(43)
Lung	Eosinophilic asthma	Pro-inflammatory	Siglec-8^+^, CD62L^+^, IL-3R^hi^; CD62L^low^ iEos positively correlates with asthma severity	—	(43, 44)

In the gastrointestinal tract, distinct functional subtypes of eosinophils have been identified. Xenakis et al. initially identified two resident eosinophil subtypes in the gastrointestinal tract, distinguished by their spatial localization and key markers (such as MHCII, CD80, CD11c): the lamina propria-associated and intraepithelial-associated subtypes (40). Larsen et al. subsequently expanded on this work, using CD11c expression intensity as a core indicator to introduce the concept of a “phenotypic continuum.” They further explored the plasticity characteristics of these subtypes in both healthy and diseased states (41). The lamina propria is predominantly composed of CD11c^−/lo^ subpopulations, characterized by high SSC and low Siglec-F/CD11b expression, indicative of resting cells. Conversely, the epithelial layer is enriched with CD11c^hi^ subsets, which exhibit decreased SSC and increased Siglec-F and CD11b expression, representing activated cells. In an ovalbumin (OVA)-induced acute allergy model, eosinophils initially displayed low CD11c expression and accumulated around crypts. Over time, these cells upregulated CD11c expression, suggesting that the local microenvironment induces eosinophil activation. A significant increase in the proportion of CD11c high-expression subsets was also observed in chronic inflammation models (41). The aforementioned research collectively established a research framework for the gastrointestinal eosinophil subtype, shifting from static classification to dynamic regulation. This advancement provides pivotal evidence for a more profound understanding of its physiological functions and pathological roles. Gurtner et al. conducted a comprehensive single-cell transcriptomic analysis of systemic eosinophils in transgenic IL-5 mice, identifying five distinct subpopulations: precursor eosinophils, immature eosinophils, circulating eosinophils, active eosinophils (A-Eos), and baseline eosinophils (B-Eos) (34). Among these, A-Eos exhibited high expression of pro-inflammatory cytokines (e.g., IL-1β, tumor necrosis factor (TNF)) and antimicrobial peptide genes (S100a family). B-Eos is widely distributed in the gastrointestinal tract, spleen, blood, and bone marrow, and shows high expression of tissue remodeling-related genes (eg, matrix metalloprotein-9, transforming growth factor-β1(TGF-β1)). A-Eos possesses antibacterial and immunomodulatory functions, capable of killing pathogenic bacteria via eosinophil extracellular DNA traps (EETs) and expressing immunomodulatory molecules such as CD80 and PD-L1. Similar activated eosinophils have also been detected in colon samples from patients with inflammatory bowel disease (IBD), where they colocalize with CD4 T cells (34).

Wang et al. utilized an esophageal epithelium and eosinophil co-culture model derived from EoE patients, in conjunction with single-cell transcriptome analysis, to identify two key eosinophil subtypes for the first time: pro-inflammatory eosinophils (EpCAM^+^CD44^+^) and pro-fibrotic eosinophils (CD69^+^IL-1R1^+^) (42). The pro-inflammatory subtype is driven by TSLP released from epithelial cells, which induces differentiation via the STAT5 pathway and expresses inflammatory factors such as CCL26 and TNF-α, thereby directly compromising epithelial barrier integrity. In contrast, the pro-fibrotic subtype relies on the activation of the IL-33/IL-1R1/NF-κB signaling axis, secretes TGF-β1 and vascular endothelial growth factor-A (VEGF-A), promotes fibroblast proliferation and collagen deposition, and is closely associated with esophageal fibrosis and stricture.

The aforementioned studies have preliminarily elucidated the molecular markers of Eos, some of which exhibit tissue-specific conservation. However, the gastrointestinal tract, as a physiologically enriched region for eosinophils, further shapes a more specific eosinophil phenotype in the microenvironment. Mesnil et al. identified two eosinophil subtypes in the mouse airway, designated as resident eosinophil (rEos) and inflammatory eosinophil (iEos). rEos, characterized by a ring-shaped nucleus, is primarily localized in the lung parenchyma, exhibiting a Siglec-F^int^CD62L^+^CD101^low^ phenotype and high expression of regulatory genes (e.g., *Anxa1, Runx3, Ldlr*). In contrast, iEos, with a lobulated nucleus, is mainly distributed in the peribronchial and bronchoalveolar lavage fluid, presenting a Siglec-F^hi^CD62L^-^CD101^hi^ phenotype and high expression of pro-inflammatory genes (e.g., *Tlr4, C3ar1, Il6*) (43). Similarly, two distinct phenotypes of eosinophils (Siglec-8^+^CD62L^+^IL-3R^low^, Siglec-8^+^CD62L^-^IL-3R^hi^) have been identified in the lungs of non-asthmatic and eosinophilic asthma patients (43). Vultaggio et al. further demonstrated that iEos with a CD62L^low^ phenotype correlates positively with asthma severity. Treatment with mepolizumab, significantly reduced the proportion of iEos while increasing the proportion of rEos (44). Unlike eosinophils in the lungs and bloodstream, intestinal eosinophils constitutively express high levels of the γ-chain (γc) (45) and lack CD62L expression (43). Compared to circulating eosinophils, intestinal eosinophils show higher expression of CD11c, SiglecF, and CD11b (40). As previously described, Gurtner et al. analyzed five eosinophil subpopulations, with A-Eos being unique to the gastrointestinal tract (34). Single-cell omics sequencing by Li et al. revealed that small intestinal eosinophils specifically express high levels of neuromedin U receptor 1 (NMUR1), which promotes degranulation and antimicrobial activity (46).

Using single-cell transcriptomics and *in vivo* fate mapping, Hu et al. established a direct link between tissue residency time and eosinophil heterogeneity in the murine gastrointestinal tract (47). Small intestinal eosinophils are long-lived, whereas colonic eosinophils are short-lived. Notably, longer residency correlates with greater heterogeneity: the small intestine harbors three transcriptionally distinct subsets—SIE1, SIE2, and SIE3—enriched in pathways of metabolic regulation, mucosal immunity, and stress/inflammation, respectively. In contrast, colonic eosinophils remain relatively uniform. Spatial analysis further reveals compartmentalization within the small intestine: long-lived, CD22^+^ specialized eosinophils (Sp-Eos) preferentially localize to villi, whereas short-lived, CD11a^+^ mature eosinophils (M-Eos) reside in crypts. Along the duodenal-to-colonic axis, CD22^+^ cells gradually decrease while CD11a^+^ cells increase, mirroring the loss of villi and expansion of crypts. These findings support a model in which niche-dependent residency time drives eosinophil diversification and spatial specialization in the gut.

The in-depth study of eosinophils’ functional subtypes offers novel insights into the pathogenesis and diagnostic and therapeutic approaches for eosinophilic gastrointestinal diseases. In EoGE, some patients may not exhibit significant peripheral blood eosinophilia or increased gastrointestinal eosinophil infiltration. This may be attributed to an imbalance in the proportion of eosinophil subtypes rather than a mere increase in eosinophil count. The identification and regulation of different eosinophil subtypes will facilitate future disease classification based on eosinophil subtypes, elucidate the variable treatment responses observed within the same disease, and guide personalized treatment strategies. Moreover, the unique characteristics of gastrointestinal eosinophils lay the foundation for more precise targeted therapies.

## 4 The multifunctional role of eosinophils in gut

**Figure 1:**
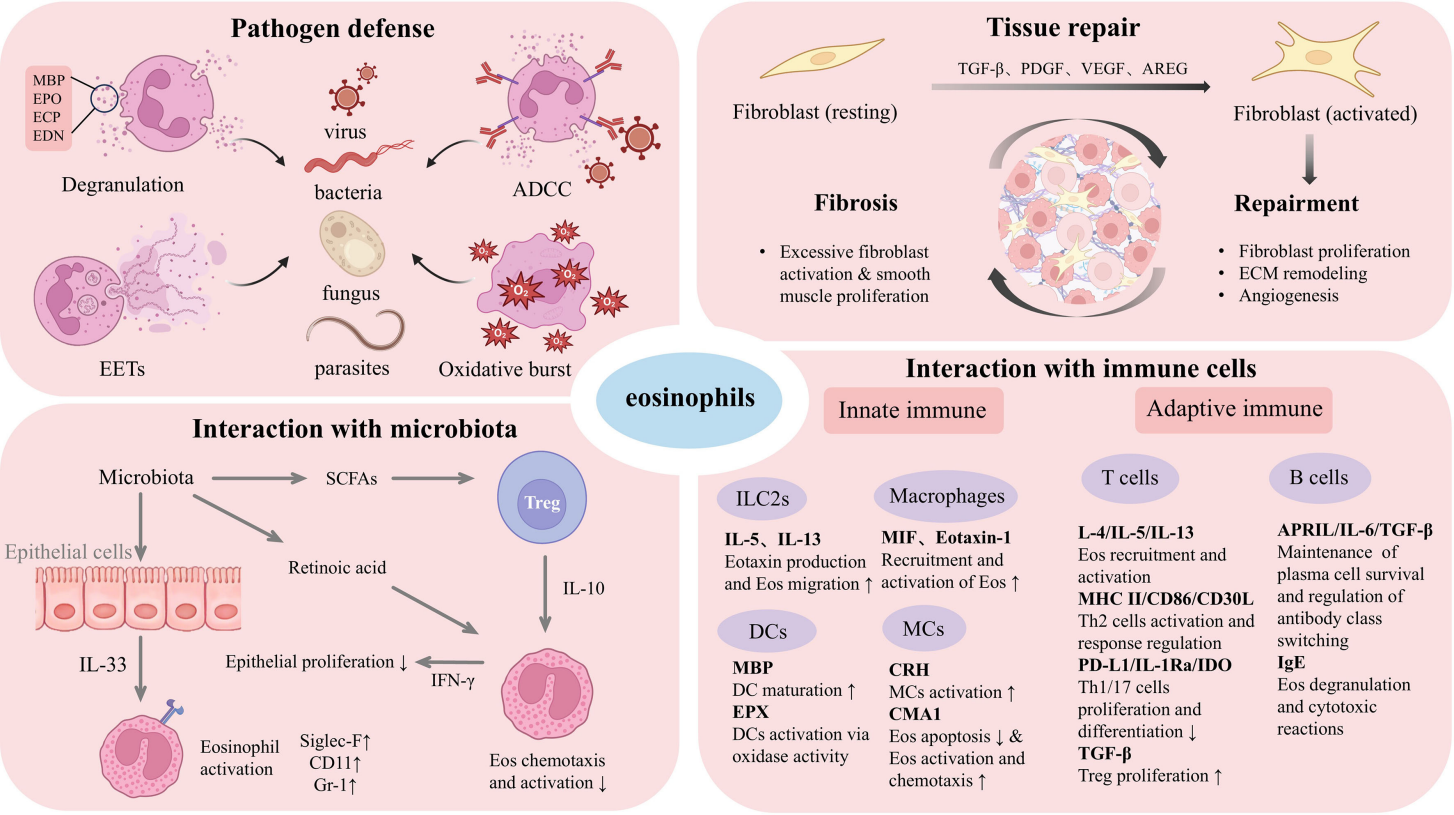
Multifunction of eosinophils in the gastrointestinal tract.

Previous studies have demonstrated that eosinophils can release a variety of cationic proteins via degranulation, mediating tissue damage upon activation in pathological states such as parasitic infections and allergic reactions, thus acting as terminal effector cells. However, as research has progressed, the role of eosinophils in maintaining homeostasis within the local immune microenvironment has been increasingly recognized. Lee et al. proposed the LIAR hypothesis (Local Immunity And/or Remodeling/Repair), which posits that eosinophils can sense diverse immunomodulatory signals in the local microenvironment and dynamically adjust their functions. These functions include promoting inflammation, maintaining homeostasis, or inhibiting inflammation and facilitating tissue repair (48). The functional diversity of eosinophils will be systematically reviewed below, with particular emphasis on their roles in pathogen defense, tissue repair, microbiota interactions, tumor microenvironment, and immune regulation (**Figure 1**).

### 4.1 Pathogen defense

When identifying pathogens such as parasites, eosinophils can not only release pre-stored intracellular granulation proteins, including MBP, eosinophil peroxidase (EPX), eosinophil cationic protein (ECP), and eosinophil-derived neurotoxin (EDN), to exert a cytotoxic effect on pathogens, but also produce large amounts of superoxide anions via the NADPH oxidase complex (49–51). These superoxide anions are subsequently converted into reactive oxygen species (ROS), such as hydrogen peroxide, which directly oxidize and damage the cellular components of parasites (52). Additionally, eosinophils express FcRγ and FcϵRII on their surface, enabling them to recognize and bind to the Fc segment of antibodies. This allows for direct action on IgG- or IgE-coated parasites via antibody-dependent cellular cytotoxicity (ADCC) (53, 54). Beyond parasitic infections, eosinophils also protect against other pathogens. In mouse models of chronic *Helicobacter pylori* infection and acute *Citrobacter rodentium* infection, Arnold et al. demonstrated that bacterial infection induces eosinophil migration to the site of infection and activation, characterized by elevated expression of CD11b and Siglec-F, along with increased EoPs in the bone marrow (55).

**Figure 2:**
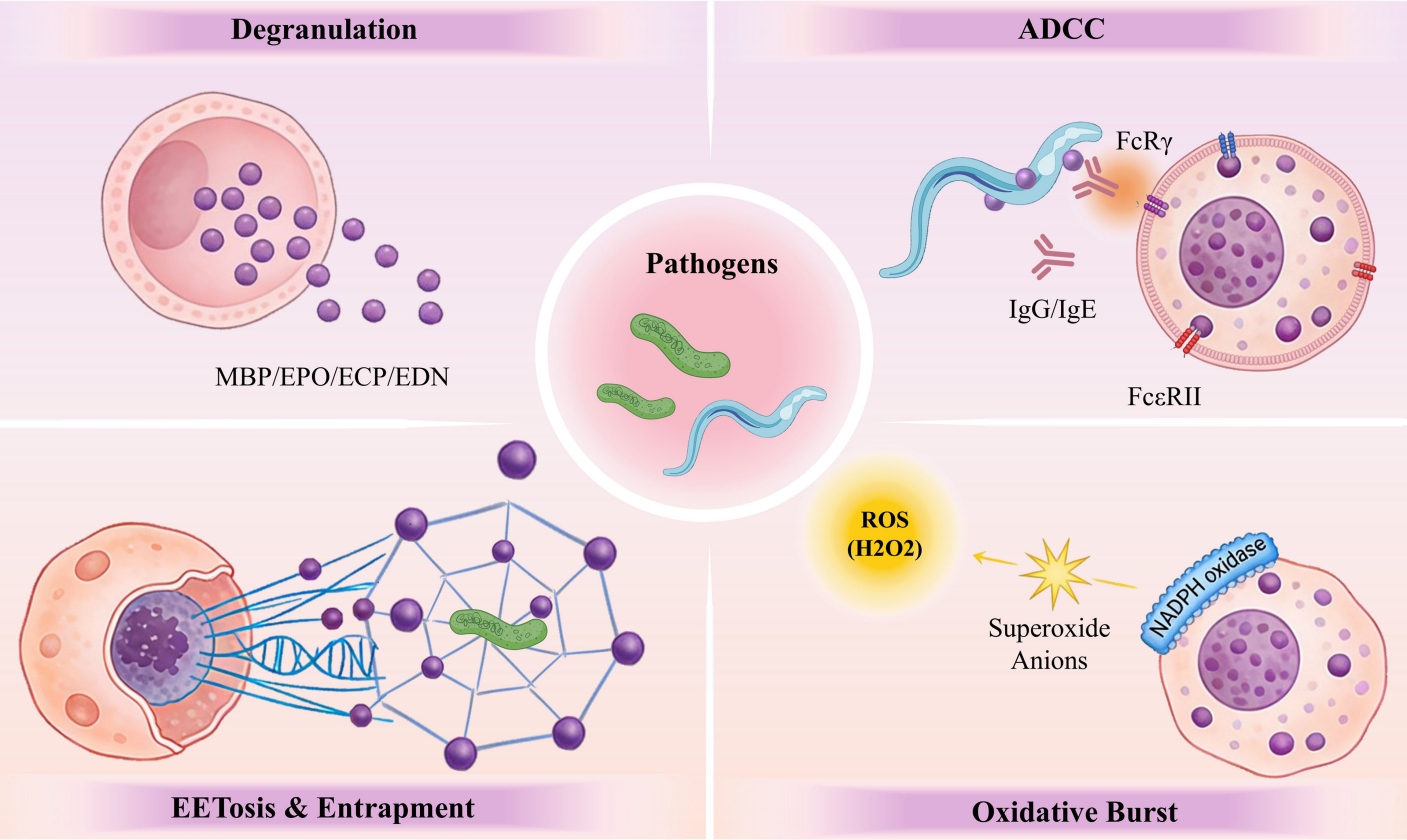
Schematic diagram of eosinophil-mediated host defense against pathogens.

Recent studies have identified EETs as an additional mechanism by which eosinophils exert their defensive functions. Ueki et al. first described eosinophil extracellular trap cell death (EETosis), a unique programmed cell death in eosinophils, which involves membrane rupture and nuclear chromatin lysis driven by reactive oxygen species produced by NADPH oxidase, leading to the release of granules and the formation of extracellular DNA networks (56). This fibrous mesh structure physically immobilizes parasites in an environment rich in toxic proteins and enzymes, thereby enhancing local killing efficacy (**Figure 2**). In a mouse model of *Citrobacter rodentium* infection, Arnold et al. observed extracellular DNA networks colocalized with EPX in the colon, confirming EET formation. Additionally, by inserting the *Diphtheria Toxin A* (*DTA*) gene downstream of an eosinophil-specific promoter to ablate eosinophils selectively, they developed an eosinophil-deficient PHIL mouse model. This model demonstrated significantly higher intestinal colonization of *Citrobacter rodentium* in transgenic line of mice (PHIL) mice compared to wild-type mice, directly highlighting the antibacterial role of EETs (55). Yousefi et al. further observed EET structures in the intestinal tissues of patients with Crohn’s disease, with immunofluorescence showing colocalization with ECP and MBP (57).

### 4.2 Gastrointestinal homeostasis maintenance

#### 4.2.1 Tissue repair

In the Th2 immune response, eosinophils can differentiate, mature, and migrate to inflammatory sites under the influence of cytokines such as IL-5 and IL-4/IL-13. They promote inflammatory responses and cause tissue damage through processes like degranulation and ROS release. According to the LIAR hypothesis, eosinophils can amplify the Th2 inflammatory response in a Th2-polarized microenvironment. Conversely, in Th1/Th17-polarized microenvironments, such as during the acute phase of infections or tumors, eosinophils can inhibit local immune responses and promote tissue repair (48). Masterson et al. demonstrated that PHIL mice exhibited more severe colonic inflammation than wild-type mice after induction with dextran sodium sulfate (DSS), trinitrobenzenesulfonic acid (TNBS), and oxazolone, highlighting the protective role of eosinophils in acute inflammation. Additionally, the production of the anti-inflammatory lipid mediator Protectin D1 was reduced in the colons of PHIL mice, and administration of exogenous protectin D1 analogues significantly alleviated colitis in PHIL mice, reducing neutrophil infiltration and the expression of pro-inflammatory factors (58).

In the inflammatory regression stage, eosinophils are regulated by local microenvironment signals (cytokines, lipid mediators) to express repair phenotypes, and participate in tissue repair, remodeling, and regeneration through the secretion of multiple mediators. During this phase, pro-inflammatory signals are attenuated, while repair signals, such as IL-10, TGF-β, and lipoxin A4, are augmented. TGF-β promotes tissue repair by stimulating fibroblast proliferation and regulating the deposition and remodeling of the extracellular matrix. Platelet-derived growth factor (PDGF) synergizes with TGF-β to facilitate tissue remodeling. Additionally, VEGF supports tissue regeneration by stimulating angiogenesis (59). α-Smooth muscle actin (α-SMA)^+^ stromal cells surrounding the crypts of the small intestine secrete GM-CSF and CCL11 after irradiation, thereby activating eosinophils and upregulating the expression of pro-fibrotic genes such as TGF-β1 (60). TGF-β1, in turn, stimulates collagen synthesis in α-SMA^+^ stromal cells, accelerating fibrosis. Hepatocyte growth factor (HGF) antagonizes the secretion of TGF-β and VEGF by eosinophils, thereby balancing the repair process and preventing excessive fibrosis and scarring (61).

**Figure 3:**
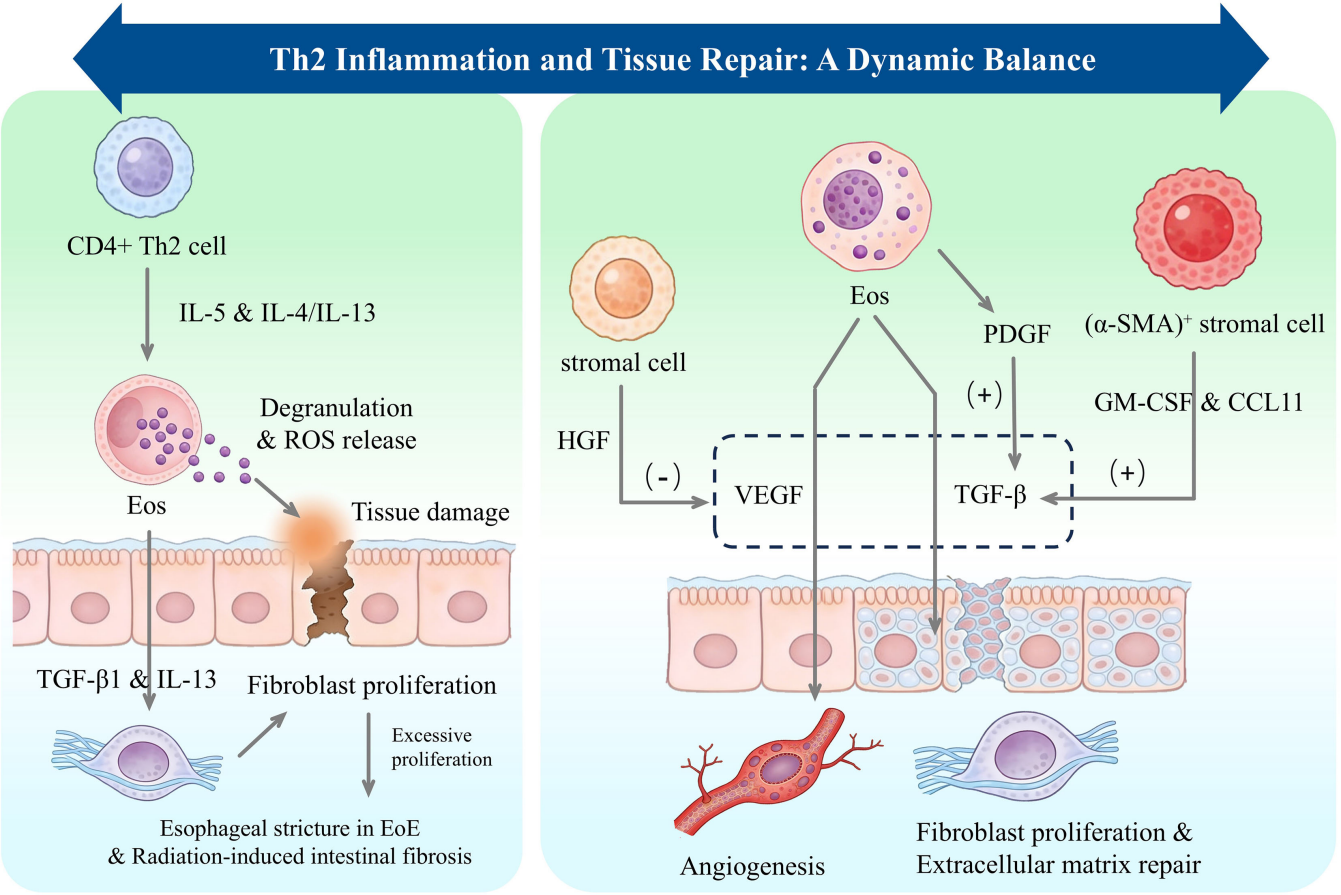
Eosinophils in Th2 inflammation and tissue repair: a dynamic balance.

The repair function of eosinophils is involved in both physiological defenses, such as after parasite clearance, and pathological inflammation, such as in eosinophilic esophagitis. However, excessive or dysregulated repair responses can result in pathological fibrosis (**Figure 3**). Regarding the contradictory role of eosinophils in gastrointestinal tissue repair and fibrosis, Doyle et al. found through analysis of eosinophilic infiltration in the esophagus of EoE patients that, during the asymptomatic phase, eosinophils primarily maintain barrier homeostasis by secreting IL-1β, IL-6, and other factors that support mucosal IgA production and epithelial renewal. Conversely, in chronic inflammation, eosinophils release toxic granule proteins that exacerbate epithelial damage. Additionally, their continuous secretion of IL-13 and TGF-β drives fibroblast activation and smooth muscle proliferation, emerging as a key driver of esophageal stricture in EoE patients (62). Takemura et al. also demonstrated that radiotherapy-induced intestinal fibrosis is characterized by substantial eosinophil infiltration and degranulation. Anti-IL-5 treatment significantly reduced intestinal eosinophil numbers and inhibited the development of radiation-induced intestinal fibrosis (RIF) in mice, highlighting the critical roles of eosinophils and IL-5 in RIF pathogenesis (60).

#### 4.2.2 Mutual regulation with microbiota

The gastrointestinal tract is colonized by a substantial population of eosinophils, one of the largest in the body, while a diverse microbiota has established ahead over a long period. Research has demonstrated that eosinophil colonization in the gastrointestinal tract occurs early in development, preceding microbial colonization (63). These two populations interact and regulate each other to maintain intestinal immune homeostasis. On one hand, the microbiota plays a central role in regulating the number, phenotype, and function of intestinal eosinophils. Germ-free (GF) mice exhibit significantly increased eosinophils in the small intestine, characterized by more cytoplasmic protrusions and fewer granules (64). Ignacio et al. compared GF mice with specific pathogen-free (SPF) mice and found that intestinal epithelial cells release IL-33 in a microbiota-dependent manner, thereby activating eosinophils. In GF mice, intestinal epithelial cells express very low levels of IL-33, and eosinophils predominantly exhibit an immature phenotype. Upon microbial recolonization, immature eosinophils will transform into a mature phenotype with the upregulation of IL-33, expressing markers such as Siglec-F, CD11b, and Gr-1, indicating that microbial signals determine baseline eosinophil levels and functional status in the gut by regulating chemotactic and survival signals (63). Cao et al. proposed that the microbiota regulates duodenal homeostasis via the retinoic acid (RA)-Eos-IFNγ axis, with *Faecalibaculum rodentium* playing a key regulatory role. This bacterium inhibits retinoate synthase expression, reduces eosinophil numbers, and diminishes the inhibitory effect on epithelial cell renewal, thereby accelerating epithelial proliferation and modulating duodenal immune homeostasis (65). On the other hand, eosinophils reciprocally shape the composition and structure of the gut microbiota. Eosinophil-deficient mice display marked gut dysbiosis, including elevated fecal bacterial loads, an imbalanced Gram-negative/Gram-positive ratio, and aberrant shifts in the relative abundances of *Firmicutes* and *Bacteroidetes*, which in turn compromise intestinal barrier integrity and host defense against pathogens (66, 67). Furthermore, the microbiota–eosinophil axis is involved in tissue remodeling and the initiation of Th2-type immunity. In the germ-free setting, eosinophil deficiency exacerbates submucosal collagen deposition and fibrosis in the intestine, while also reducing food antigen-specific IgE and IgG1 production, suggesting that the microbiota modulates intestinal repair and susceptibility to food allergy via eosinophils (64). Conversely, eosinophils can directly sense commensal and probiotic signals through pattern recognition receptors such as TLR2 and NOD1/2, releasing antimicrobial proteins and anti-inflammatory factors to fine-tune the intestinal microenvironment (67).

Thus, the microbiota and intestinal eosinophils do not merely interact but functionally co-adapt, forming a homeostatic loop in which microbial signals license eosinophil maturation and effector readiness, while eosinophils reciprocally calibrate microbial community structure and immune tone. This reciprocal circuit extends beyond local regulation to influence tissue remodeling, allergic priming, and possibly disease susceptibility. Unraveling how this bidirectional axis is tuned in different intestinal niches and disrupted in pathology may open new avenues for microbiota- or eosinophil-targeted interventions in gastrointestinal disorders.

#### 4.2.3 Interaction with immune cells

Whether they colonize the gastrointestinal tract in large numbers under steady-state conditions or abnormally proliferates and becomes activated under pathological conditions such as parasitic infections and allergic reactions, the role of eosinophils involves interactions with a variety of immune cells. These interactions help maintain gastrointestinal immune homeostasis under physiological conditions but may become significantly dysregulated in pathological states. Understanding the interactions between eosinophils and other immune cells may provide new directions for targeted interventions in gastrointestinal eosinophilic diseases.

##### 4.2.3.1 Innate immune system

The gastrointestinal tract harbors a complex immune barrier. Innate immune cells, including macrophages, MCs, and ILC2, form a crucial part of this barrier. Gastrointestinal eosinophils interact with these intrinsic immune cells to participate in homeostasis maintenance and immune regulation.

The interaction of eosinophils and macrophages has been indicated by many studies. Ignacio et al. reported no significant difference in the number of monocytes/macrophages in the small intestine of ΔdblGATA-1 mice compared to healthy controls. However, they observed an increase in mature Ly6C^-^MHCII^+^ macrophages in the lamina propria of the small intestine following bone marrow-derived eosinophil transplantation, suggesting that eosinophils regulate macrophage maturation and survival (63). Yang et al. first proposed that eosinophils can modulate macrophage-driven hepatocyte regeneration via the IL-4/heparin-binding epidermal growth factor (HB-EGF) axis. In a mouse model of liver ischemia-reperfusion injury, eosinophils infiltrate the liver and secrete IL-4, which binds to the IL-4Rα on macrophages, inducing HB-EGF expression and thereby promoting hepatocyte proliferation and repair (68). Macrophages can also promote the recruitment and activation of eosinophils by expressing macrophage migration inhibitory factor (MIF), CCL11, etc. MIF, secreted by various cells including macrophages, Th2 cells, and eosinophils, exerts pro-inflammatory effects in Th2 inflammation by binding to receptors such as CD74, CXCR2, and CXCR4 (69–71). In type 2 inflammation, MIF directly promotes eosinophil chemotaxis. Souza et al. demonstrated that in EoE, MIF promotes eosinophil migration and esophageal tissue remodeling through CXCR4-dependent mechanisms (72). Ahrens et al. demonstrated that intestinal CD68^+^ macrophages secrete eotaxin-1, thereby promoting eosinophil recruitment in pediatric UC patients (73). Waddell et al. further identified CCR2^-^Ly6C^hi^ monocytes/macrophages as the primary source of CCL11 in DSS-induced mouse colitis, with eosinophil recruitment depending on CCR2 (74). Lampinen et al. validated that CD14^+^CD33^+^ myeloid cells regulate eosinophils via CCL11 in adult patients, noting that this axis remains active even in quiescent UC, suggesting its role in disease maintenance and recurrence (75). At the signal transduction level, both NF-κB and STAT-6 pathways were found to be involved in macrophage activation in DSS-induced colitis in mice. However, STAT-6 knockout mice still exhibited macrophage recruitment, elevated CCL11, and eosinophil infiltration under DSS-induced conditions, indicating that STAT-6 may be a non-essential pathway, while NF-κB likely plays a predominant role (76).

MC activation is a critical component in many allergic and inflammatory diseases. These cells express CCR3 to varying degrees, which can be activated by chemokines such as eotaxin and RANTES, facilitating their migration into inflammatory sites and differentiation (77). In allergic reactions, the classic mechanism involves IgE binding to high-affinity receptors (FcϵRI) on the MC surface, triggering rapid activation and degranulation. This process releases histamine, leukotrienes, and other mediators, leading to increased vascular permeability and smooth muscle contraction, which constitutes the immediate reaction. Subsequently, within 4–24 hours, eosinophils and neutrophils are recruited to the site of inflammation, contributing to tissue damage and chronic symptoms (78). Wong et al. demonstrated that in allergic diseases, chymase (CMA1) secreted by MCs can inhibit eosinophil apoptosis, induce the expression of chemokines like IL-6 and CXCL8, upregulate the adhesion molecule CD18, and promote eosinophil activation and chemotaxis (79). Conversely, eosinophils can also activate MCs. Research has shown that psychological stress can exacerbate intestinal inflammation via the “EOS-corticotropin-releasing hormone (CRH)-MC” axis, where eosinophil-derived CRH activates MCs, thereby inducing dysfunction of the jejunal epithelial barrier (80). Similar to eosinophils, MCs have also been identified to possess functional subtypes that represent both steady-state and activated conditions. Based on the types of enzymes released through degranulation, MCs can be categorized into two subgroups: MC_T_, which secrete tryptase only, and MC_TC_, which secrete tryptase (TPSAB1), CMA1, carboxypeptidase A3 (CPA3), and others (81). In patients with EoE, Morgenstern et al. more precisely delineated the MC subsets via single-cell sequencing. Resident MCs are primarily located in the lamina propria, expressing TPSAB1 and amphiregulin (AREG), and maintaining epithelial homeostasis. In contrast, activated MCs are mainly found in the epithelial layer and can be further divided into transient and persistent MCs, depending on their detectability during remission. Persistent MCs continue to express high levels of MC activation-related genes, such as *CSTG* and *CMA1*, even during remission, suggesting their potential involvement in disease recurrence (82).

ILC2s are predominantly located on the surfaces of skin and mucous membranes. They are the primary cells expressing IL-5 in non-lymphoid tissues such as the lungs and intestines under steady-state conditions (83). First identified by Neill et al., ILC2 is activated via the IL-25 and IL-33 pathways and exhibits high expression of IL-13 and IL-5 (84). IL-25, derived from tuft cells, activates ILC2 to secrete IL-13, which acts on epithelial crypt progenitor cells, promoting the differentiation of goblet and enteroendocrine cells and mediating type 2 immunity-associated epithelial remodeling in the small intestine (85). ILC2, activated by IL-33, proliferates extensively and expresses AREG, which binds to epidermal growth factor receptor (EGFR) on epithelial cells, thereby promoting epithelial cell proliferation and differentiation, enhancing mucosal barrier function, and limiting inflammatory responses while promoting tissue repair, with potential therapeutic applications in IBD (86). ILC2 is essential for eosinophil differentiation and homing under steady-state conditions, and ILC2-deficient mice exhibit significantly reduced eosinophil numbers in the bone marrow, blood, lungs, and intestinal tissues (87). ILC2 can also induce eosinophil activation and migration through various pathways. For example, during helminth infection, ILC2s are activated and co-express IL-13, promoting local eotaxin production and inducing eosinophil migration to sites of inflammation (83). Mice lacking aryl hydrocarbon receptors (AHRs) in eosinophils exhibit expanded type 2 innate lymphocytes and more effectively clear *Nippostrongylus brasiliensis* infection compared to wild-type mice. These findings underscore the heterogeneity of eosinophil responses to tissue-specific cues and identify a unique AHR-dependent eosinophil subset with immunomodulatory properties in the small intestine (88).

In summary, gastrointestinal eosinophils engage in reciprocal crosstalk with macrophages, mast cells, and ILC2s to coordinate intestinal homeostasis and inflammatory responses. Eosinophils facilitate macrophage maturation and IL-4/HB-EGF-mediated tissue repair, whereas macrophages recruit and activate eosinophils via MIF and CCL11. Reciprocal activation occurs between eosinophils and mast cells, with eosinophil-derived CRH compromising epithelial barrier integrity and mast cell-derived chymase promoting eosinophil chemotaxis. ILC2s regulate eosinophil differentiation, homing, and activation through IL-5, IL-13, and AREG signaling. Collectively, these integrated networks position eosinophils as central regulators within the innate immune landscape of the gastrointestinal tract.

##### 4.2.3.2 Adaptive immune system

The adaptive immune system equips the gastrointestinal tract with immune tolerance and specific defenses against pathogens and food allergens. Eosinophils contribute to diverse adaptive immune responses through interactions with T cells, B cells, and DCs.

**Figure 4:**
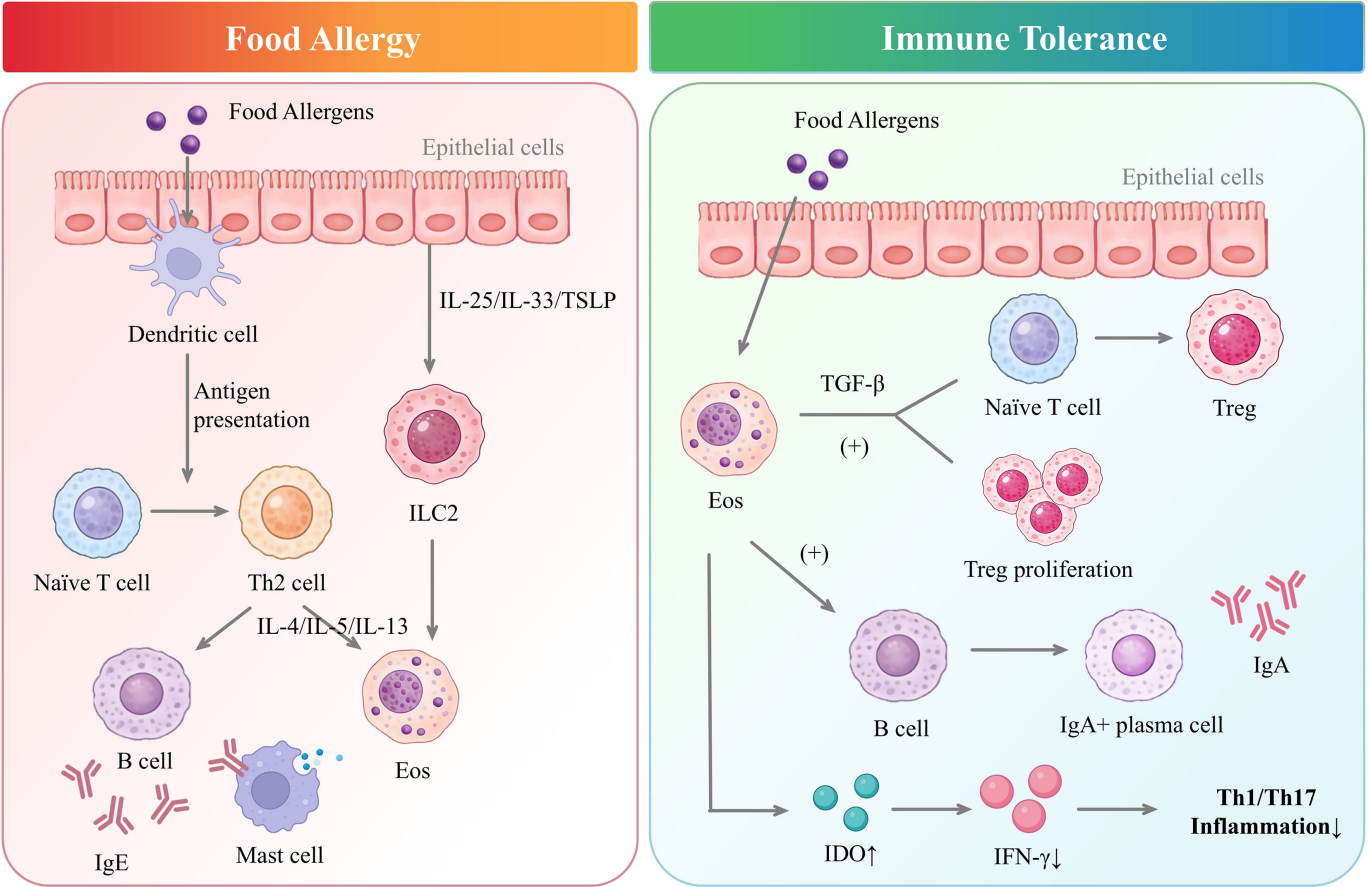
Schematic diagram of eosinophil involvement in food allergy and immune tolerance.

T cells are central drivers of gastrointestinal eosinophil infiltration, with CD4^+^Th2 and CD8^+^Tc2 cells secreting IL-4, IL-5, and IL-13 to recruit and activate eosinophils (89). Ramon et al. demonstrated a close interaction between T cells and eosinophils in Nedd4 family interacting protein 1 (Ndfip1)-deleted mice, where T cell overactivation and elevated IL-5 levels promote eosinophil infiltration into the gastrointestinal mucosa. This T cell activation precedes eosinophil infiltration, confirming that T cells regulate eosinophil recruitment via cytokine secretion (90). Beyond their destructive effects, eosinophils also maintain local immune homeostasis by modulating T cell activity. Recent studies have shown that intestinal A-Eos express MHC II and costimulatory molecules (e.g., CD86, CD30L), which can activate Th2 cells and regulate their responses (91, 92). In IBD patients, A-Eos numbers are significantly increased and colocalized with CD4 T cells, highlighting their role in pathology. Interestingly, eosinophils can also express PD-L1, which activates eosinophils while preventing excessive immune responses and maintaining immune balance (34). Arnold et al. found that gastrointestinal eosinophils directly inhibit Th1 cell proliferation and cytokine secretion (e.g., IFN-γ) through IFN-γ-dependent PD-L1 upregulation. In PHIL mice and anti-IL-5 antibody-treated models mimicking eosinophil deficiency, Th1 responses are overactivated, leading to increased gastrointestinal inflammation and impaired bacterial clearance (55). In addition to Th1 cells, eosinophils regulate Th17 cells through IL-1 receptor antagonists (IL-1Ra) encoded by high expression of *Il1rn*, which is another key mechanism for maintaining intestinal homeostasis. Sugawara et al. found in a small bowel homeostasis model that small intestinal eosinophils can secrete IL-1Ra, which blocks IL-1β activation signals to Th17 cells by competitively binding to IL-1 receptors, preventing an excessive inflammatory response. Deletion of eosinophils (ΔdblGATA-1 mice) results in a significant increase in the number of Th17 cells in the small intestine, while IL-1Ra-deficient eosinophils completely lose their inhibitory ability to Th17 (93). These studies demonstrate that eosinophils can inhibit the Th1/Th17 response in the gut to maintain immune homeostasis in a steady state. The regulatory mechanisms of T cells by human eosinophils are highly similar to those observed in animal models. Odemuyiwa et al. showed that human peripheral blood and lung-infiltrating eosinophils can constitutively express indoleamine 2,3-dioxygenase (IDO), which catalyzes the metabolic conversion of tryptophan to kynurenine. This process inhibits Th1 differentiation by reducing IFN-γ secretion, leading to a Th2 bias (94). This process also plays an essential role in intestinal immune tolerance (**Figure 4**). In addition, regulatory T cells (Tregs), as core regulators of immune homeostasis, form a complex interaction network with eosinophils to jointly limit excessive inflammatory responses. Under physiological conditions, eosinophils in the intestinal Peyer’s patches (PP) induce the differentiation of naïve CD4^+^ T cells into Tregs by secreting RA and TGF-β. They also promote the generation of “RORγt^+^ antigen-presenting cells” (such as ILC3s), thereby maintaining immune tolerance to food antigens and commensal flora (95). This function is unique to intestinal eosinophils (**Figure 4**). In the presence of bacterial infection or allergen stimulation, eosinophil-expressed TGF-β specifically promotes the proliferation of RORγt^+^Foxp3^+^ Tregs without inducing conventional T cell expansion (96). *Tgfb* gene ablation or Treg deficiency can lead to excessive T cell responses biased toward Th2 polarization, exacerbating inflammation.

Eosinophils play a crucial role in humoral immunity, regulating B cell activation, plasma cell survival, and antibody production, particularly in gastrointestinal mucosal immunity. Wong et al. demonstrated that eosinophils directly modulate B cell homeostasis. In transgenic mice (NJ1638) with high IL-5 expression, B cells were significantly increased, whereas in NJ1638 PHIL mice with high IL-5 levels but lacking eosinophils, B cell expansion was absent. *In vitro* studies revealed that human eosinophils promote B cell survival, proliferation, and immunoglobulin secretion via a non-contact mechanism (soluble factors) independent of eosinophil activation status (97). Beyond regulating peripheral B cell numbers, eosinophils also drive the differentiation of mucosal B cells into IgA^+^ plasma cells, which is crucial for inducing immune tolerance and maintaining intestinal immune homeostasis (**Figure 4**). In the murine intestinal lamina propria, eosinophils closely colocalize with IgA^+^ plasma cells, and the secretion of activation and proliferation-induced ligand (APRIL), IL-6, and active TGF-β by eosinophils is essential for IgA class switching and plasma cell survival (98). Chu et al. found that activation of eosinophils with lipopolysaccharide (LPS) *in vitro* and with antigens *in vivo* significantly upregulated the expression of plasma cell survival factors such as APRIL, IL-6, IL-4, IL-10, and TNF-α, indicating that eosinophils maintain long-term plasma cell survival in the bone marrow (99). In eosinophil-deficient mice, the production and maintenance of IgA plasma cells are impaired, leading to decreased serum and intestinal secretory IgA levels. This is accompanied by inhibited IgA class conversion in PP (100). Jung et al. reported significant reductions in IgA levels, decreased intestinal mucus secretion, and PP dysplasia in various eosinophil-deficient mouse models (101). However, RNA microarray analysis revealed no significant changes in the expression of APRIL, B-cell activating factor of the TNF family (BAFF), and TGF-β in intestinal eosinophils. This discrepancy with the findings of Chu et al. may be attributed to the CD11b^+^SiglecF^+^ strategy employed by Chu et al. for eosinophil screening, which did not exclude potential confounding by CD11b DCs. Further studies have elucidated the molecular mechanisms by which eosinophils promote IgA class conversion. Gastrointestinal eosinophils highly express IL-1β, which activates ROR-γt^+^ ILCs to secrete LTα/β, while upregulating iNOS expression. iNOS, an enzyme regulating IgA class switching, facilitates IgA class conversion. IL-1β-deficient mice recapitulated the phenotype of eosinophils deletion, characterized by reduced IgA^+^ cells and impaired PP development, confirming IL-1β as a key effector molecule in eosinophil regulation of intestinal B cells (101). When foreign antigens trigger a Th2 immune response, B cells are activated by Th2 cells and by IL-4 and IL-13 secreted by activated eosinophils. This activation induces class switching from IgM to IgE, followed by differentiation into plasma cells that synthesize and secrete large amounts of IgE. IgE, in turn, regulates eosinophils through positive feedback, binding to FcϵRI on their surface to induce degranulation and cytotoxic reactions (102). Varela et al. demonstrated that in a mouse model of allergic enteritis induced by OVA, mice overexpressing IgE exhibited more severe intestinal inflammation, characterized by increased eosinophil infiltration and alterations in the fecal microbiome, suggesting a synergistic effect of elevated IgE and eosinophils in gastrointestinal eosinophilic diseases (103). Beyond promoting antibody class switching, eosinophils can also inhibit excessive switching and maintain the balance of antibody class switching. In mice infected with *Heligmosomoides polygyrus*, eosinophils maintain IgA dominance by restricting IL-4 secretion from T follicular helper (TFH) cells in the PP, thereby preventing excessive conversion of B cells to IgG1 (104). However, recent studies have shown that eosinophils are not essential for maintaining homeostatic IgA levels. FitzPatrick et al. used a littermate control strategy to systematically compare wild-type and eosinophil-deficient (dblGATA) mice, finding no significant differences in intestinal secretory IgA levels, small intestinal IgA plasma cell numbers, or circulating IgA levels between the two groups (105). Beller et al. demonstrated that the low IgA phenotype in eosinophil-deficient mice could be reversed by co-housing with mice having high IgA levels, with the core mechanism involving gut microbiota exchange—specifically, *Anaeroplasma* spp. bacteria inducing TGF-β secretion by TFH cells in Peyer’s patches, thereby promoting IgA class switching. When these bacteria were introduced into deficient mice via fecal microbiota transplantation, their IgA levels significantly increased, independent of eosinophil presence (106).

DCs serve as the critical bridge between innate and adaptive immunity, functioning as the most potent antigen-presenting cells. They are capable of antigen uptake, T cell activation, and regulation of downstream Th1/Th2 immune responses. In response to alarm signals such as TSLP released by damaged epithelium, DCs can maintain Th2 central memory T cells (T_CM_s) and further amplify and polarize these T_CM_s via the OX40/OX40L pathway (107). Another alarm hormone, IL-33, has also been shown to activate DCs and drive Th2 polarization (108). During Th2 inflammation, the maturation, activation, and migration of DCs may also depend on eosinophil activity. A study of African catfish (*Clarias gariepinus*) naturally infected with worms revealed a significant increase in intestinal eosinophil granulocytes, accompanied by the aggregation and activation of CD68^+^ DCs. The infiltration of eosinophils and DCs was closely associated with the upregulation of proliferation and angiogenesis-related molecules such as proliferating cell nuclear antigen (PCNA) and VEGF (109). This suggests that in the fish gut, eosinophils synergize with immune cells like DCs in anti-parasitic infections. Lotfi et al. found that eosinophils can induce DC maturation (110). *In vitro* studies utilized CpG Class C oligodeoxynucleotide (CpG-C) to mimic pathogen stimulation, thereby activating the TLR-9 pathway. This activation induced eosinophil degranulation and the subsequent release of MBP. Confocal microscopy revealed that MBP was internalized by DCs, concurrent with significant upregulation of DC maturation markers, including CD80, CD83, CD86, and HLA-DR. In contrast, no similar effects were observed in neutrophils (110). EPO, akin to MBP, is pivotal in the interaction between eosinophils and DCs. Derek et al. demonstrated that EPO released upon eosinophil activation directly activates murine and human DCs via its oxidase activity. This activation promotes the expression of costimulatory molecules and chemokine receptors, such as CD80, CD86, and CCR7, while inhibiting IL-12p40 production, thereby fostering a microenvironment conducive to Th2 polarization (111).

Collectively, gastrointestinal eosinophils intricately coordinate adaptive immune responses through bidirectional interactions with T cells, B cells, and DCs. They support Th2 responses via MHC II and costimulatory molecules while restraining Th1/Th17 activity through PD-L1 and IL-1Ra, thereby preserving intestinal immune homeostasis. Eosinophils further promote IgA class switching and plasma cell survival via APRIL, IL-6, and IL-1β, though their essentiality for basal IgA production may depend on microbial context. Additionally, eosinophil-derived MBP and EPO induce DC maturation and Th2-biased polarization. In summary, gastrointestinal eosinophils exhibit complex bidirectional regulation of innate and adaptive immune cells within their microenvironment, participating in both inflammatory responses and immune homeostasis. Understanding the interactions between eosinophils and these immune cells can enhance our comprehension of the pathogenesis of gastrointestinal eosinophilic diseases and identify novel therapeutic targets.

#### 4.2.4 Eosinophils in tumor microenvironment

Recent evidence positions eosinophils as active anti-tumoral players in the gastrointestinal tumor microenvironment, particularly in colorectal cancer (CRC). Large-scale automated quantification of 1,625 CRC patients revealed that eosinophils at the tumor front (EosF) independently predict favorable prognosis (HR = 0.70), with stronger effect in microsatellite-instable cases (HR = 0.32) (112). Notably, EosF and intraepithelial lymphocytes provide additive prognostic information, suggesting eosinophils operate through mechanisms distinct from adaptive immunity (112). A meta-analysis of 6,384 patients further confirmed that tumor-associated tissue eosinophilia correlates with improved overall survival in CRC and esophageal carcinoma, and inversely associates with lymph node metastasis and advanced stage (113).

Mechanistically, eosinophils exert direct tumoricidal activity through degranulation, ETosis, and contact-dependent cytotoxicity (114, 115). Beyond these effector functions, they promote antitumor immunity via cross-talk with T cells, primarily through the GM-CSF–IRF5 signaling axis. In murine colorectal cancer models, eosinophil depletion impaired intratumoral IFN-γ and TNF-α production by CD4^+^ and CD8^+^ T cells and accelerated tumor growth, whereas eosinophil-specific deletion of *Csf2rb*, *Csf2ra*, or *Irf5* recapitulated this defect. Conversely, IL-10 produced by tumor-associated myeloid cells suppressed GM-CSF-induced IRF5 phosphorylation and eosinophil activation, and eosinophil-specific loss of IL-10 receptor or STAT3 enhanced antitumor immunity. In human colorectal cancer, high eosinophil infiltration correlates with improved survival and co-localizes with CD8^+^ T cells (116). Moreover, peripheral eosinophilia has emerged as a potential biomarker for response to immune checkpoint inhibitors in gastrointestinal malignancies, with higher eosinophil counts linked to better treatment outcomes. Collectively, these findings support the incorporation of eosinophil-related parameters into prognostic models and immunotherapeutic strategies.

Thus, eosinophils exert dual anti-tumoral functions in the gastrointestinal tumor microenvironment, particularly in colorectal cancer. They directly eliminate tumor cells through degranulation, ETosis, and contact-dependent cytotoxicity, and indirectly enhance antitumor immunity via the GM-CSF–IRF5 signaling axis that promotes Th1 and CD8^+^ T cell responses, a process counter-regulated by IL-10. Clinically, high eosinophil infiltration predicts favorable prognosis and correlates with better response to immune checkpoint inhibitors, supporting their integration into prognostic models and immunotherapeutic strategies.

## 5 Cross-tissue modulation of eosinophils

Abnormal infiltration of eosinophils in various tissues can cause organ-specific or systemic pathological damage, as seen in asthma (affecting the airway and lung), EoGE (affecting gastrointestinal tract), hypereosinophilic syndrome (multisystem involvement), eosinophilic granulomatosis with polyangiitis (multisystem involvement), and other diseases. Despite their diverse presentations, these conditions always involve cross-tissue migration of eosinophils and disruption of local homeostasis. Recent studies have further demonstrated that eosinophil function is regulated across multiple organs and systems.

**Figure 5:**
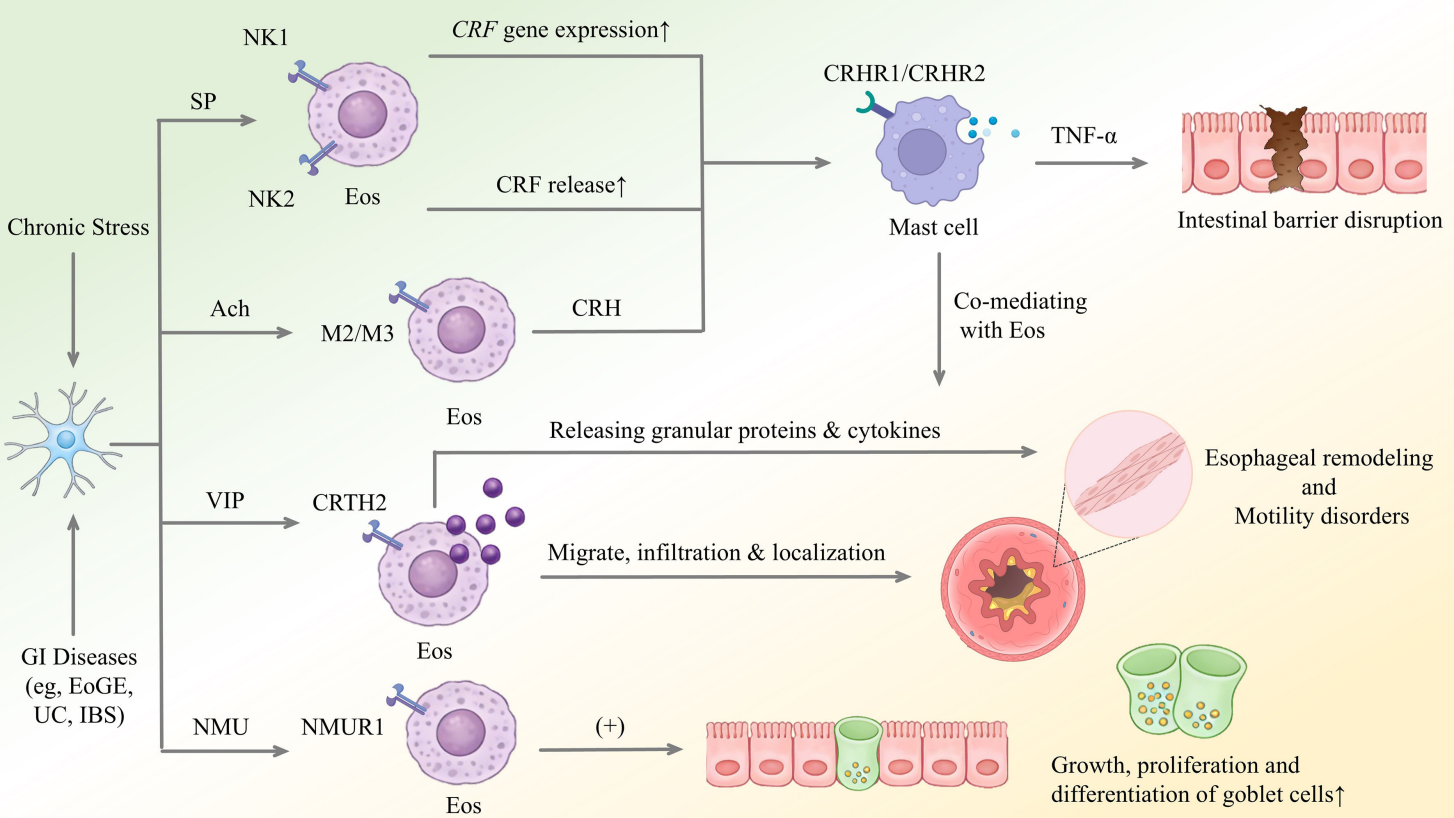
Schematic illustration of eosinophil-mediated neuroimmune regulation in the gastrointestinal tract.

The gastrointestinal tract, a highly innervated immune tissue, harbors eosinophils that can be regulated by the nervous system to maintain immune homeostasis and respond to immune signals (**Figure 5**). Li et al. identified unique transcriptomic features of small intestinal eosinophils, including significant expression of the NMUR1, through single-cell sequencing of eosinophils from multiple tissues (46). Organoid co-culture experiments demonstrated that NMU-stimulated eosinophils significantly enhanced organoid growth and the proliferation and differentiation of goblet cells. Furthermore, precise activation of NMU neurons using the Designer Receptors Exclusively Activated by Designer Drugs (DREADDs) system in mice led to a substantial increase in intestinal goblet cells, indicating that NMU can indirectly regulate intestinal epithelial homeostasis via eosinophils.

Psychological stress is implicated in the pathogenesis of many intestinal diseases. Zheng et al. demonstrated that chronic stress significantly upregulates CRH expression in murine intestinal eosinophils, with a positive correlation to stress duration (80). In EoGE, Kanamori et al. used a water avoidance stress (WAS) model to show that stress markedly exacerbates OVA-induced intestinal inflammation. Immunofluorescence revealed co-localization of MCs and CRH-R1 in the ileum, and quantitative RT-PCR confirmed significant upregulation of *Crh* mRNA. Pre-treatment with a CRH-R1 antagonist significantly mitigated the WAS-induced effects, highlighting the role of the Eos-CRH-MC axis in stress-mediated intestinal inflammation (117). In UC, Wallon et al. elucidated that intestinal barrier disruption involves neuronal-immune interactions, where cholinergic nerve signals activate eosinophils via M2 and M3 receptors, prompting CRH release. This, in turn, activates neighboring MCs, leading to the release of mediators like TNF-α that compromise the epithelial barrier (118). In irritable bowel syndrome (IBS), increased jejunal eosinophils with a degranulated phenotype were observed, with significantly higher CRH content compared to healthy controls. This CRH content correlated positively with clinical symptoms, stress, and depression scores. *In vitro* studies confirmed that substance P and carbacholine stimulation induce CRH secretion from eosinophils within 30 minutes without triggering the release of inflammatory proteins like MBP (119).

In addition to local immune responses, eosinophils can also participate in the cross-layer regulation of gastrointestinal smooth muscle function in response to neurogenic signals. Yadavalli et al. demonstrated that in EoE, eosinophils can respond to vasoactive intestinal peptides (VIPs) secreted by nerve cells. Through their surface CRTH2 receptors, eosinophils migrate across tissues, infiltrate from the esophageal epithelial layer to the muscle layer, and localize adjacent to muscle nerve cells. Here, they co-mediate esophageal remodeling and motor dysfunction with MCs via the release of granular proteins and cytokines. CRTH2 antagonists can reduce the cross-tissue infiltration of eosinophils and improve esophageal smooth muscle movement abnormalities, revealing a key mechanism by which neural signals regulate the cross-tissue effects of eosinophils (120).

Recent studies have demonstrated that intestinal eosinophils can amplify systemic inflammation and contribute to the progression of diseases in other organs or systems. An increasing number of investigations suggest that disturbances in the gut microbiota and its metabolism are key drivers in the onset and exacerbation of type 2 inflammation. Burrow et al. identified a gut symbiotic protozoan, *Tritrichomonas musculis* (T.mu), which stimulates goblet cells to release IL-25, thereby activating ILC2 and inducing their migration to the lungs, leading to eosinophil accumulation and worsening of allergic diseases such as asthma (121). Kanj et al. found that mice with intestinal *Candida albicans* dysregulation exhibited more severe asthma symptoms, increased peripheral blood IgE, and airway eosinophil infiltration following sensitization with house dust mite (HDM); these mice also had higher ILC2 levels in lung tissue, suggesting an ILC2-mediated mechanism (122). Arifuzzaman et al. demonstrated that an inulin fiber diet upregulates microbiome-derived bile acids and promotes eosinophil infiltration in the intestines and lungs of mice via the Farnesoid X receptor (FXR)-IL-33-ILC2 axis (123). Conversely, certain probiotics can modulate eosinophil activity in the gastrointestinal tract and lungs through the gut-pulmonary axis, exerting anti-inflammatory effects. For instance, *Bifidobacterium breve* (MRx0004) reduces eosinophil recruitment by lowering pulmonary pro-inflammatory factors (CXCL2, IL-1β), while *Lactobacillus fermentum* inhibits eosinophil-mediated airway inflammation by increasing short-chain fatty acids (SCFAs), maintaining intestinal barrier integrity, and activating FOXP3^+^Tregs. These findings highlight that gut microbiota metabolites and immunomodulation are crucial mechanisms for eosinophil cross-tissue regulation (124, 125). Thus, modulating the gut microbiota or its metabolites may represent a potential therapeutic strategy for allergic pulmonary diseases. Yip et al. found that oral or maternal supplementation of butyrate significantly improved the high airway response, eosinophil infiltration, and IgE levels induced by OVA/HDM in mice (126, 127). Butyrate is a type of SCFAs, which can act as a histone deacetylase (HDAC) inhibitor and exert an anti-inflammatory effect in asthma through epigenetic regulation, such as promoting eosinophil apoptosis, inhibiting CCR3 expression to curb eosinophil chemotaxis (128), suppressing FcϵRI signaling to reduce MC degranulation (129), and facilitating extrathymic differentiation of Tregs (130), etc. Lai et al. found that asthma induced intestinal dysbiosis in rats, while fecal bacterial transplantation treatment in healthy controls improved the inflammatory response in asthmatic rats and corrected SCFA dysregulation in the gut (131). Guo et al. found that rosmarinic acid (RosA) can improve asthma by regulating metabolites of the gut microbiome, and RosA can inhibit the production of LPS while upregulating SCFA to enhance intestinal barrier function and prevent eosinophils from migrating to the blood and lung tissue. RosA can also reduce the synthesis of 5-hydroxytryptamine (5-HT) in the gut and inhibit bronchoconstriction and vasodilation. The gut microbiota of mice treated with RosA can reproduce the anti-asthmatic effect, further confirming the importance of the gut-pulmonary axis (132).

Similar interactions of eosinophils have been observed in the skin and intestines. Kim et al. reported that psoriasis patients exhibit elevated markers of intestinal barrier damage, with small intestine transcriptomics revealing enrichment of pathways such as *TLR8/9* and *C1QB/C*, which regulate phagocytosis and degranulation (133). In an imiquimod-induced psoriatic dermatitis mouse model, TLR7 signaling was activated, triggering eosinophil degranulation in the small intestine and subsequent intestinal barrier damage. Bone marrow eosinophils from these mice, when transfused into eosinophil-deficient ΔdblGATA mice, could replicate imiquimod-induced skin lesions, suggesting a bidirectional pathogenesis between the small intestine and skin (133). Additionally, targeting the gut microbiota can ameliorate cutaneous atopic diseases. Zhao et al. and Kim et al. demonstrated that *Lactobacillus reuteri* and *Lactobacillus paracasei*, respectively, can mitigate atopic dermatitis symptoms and reduce skin eosinophil infiltration by promoting the proliferation of CD4^+^CD25^+^Foxp3^+^ Tregs and modulating the gut microbiota (134, 135). Fang et al. demonstrated that *Bifidobacterium adolescentis* promotes Treg differentiation, inhibits the Th2 response, and increases the proportion of lactic acid bacteria in mice, thereby alleviating symptoms of specific dermatitis (136). Similarly, *Kazachstania turicensis* CAU Y1706 ameliorated atopic dermatitis by modulating the gut-skin axis, highlighting that not only bacteria but also fungal microorganisms can regulate eosinophils within the intestinal axis, thereby expanding the diversity of microbial interventions (137). Zhang et al. found that a mixture of *Schizonepeta tenuifolia* and *Alpinia oxyphylla* also relieved atopic dermatitis and improved the gut microbiome in NC/NGA mice. These findings suggest that plant extracts, in addition to microorganisms, can serve as an alternative strategy for modulating the gut-skin axis, with the mixture showing superior efficacy (138).

The study by Olbrich et al. further elucidated the interactive and mutually reinforcing relationship among the lung, skin, and intestine. Specifically, local stimulation of these organs with OVA led to varying degrees of eosinophilia and activation phenotypes in distant organs. Moreover, mice with prior intestinal or skin exposure to OVA exhibited more pronounced airway allergic inflammation in response to subsequent HDM inhalation (139).

Despite significant advancements in understanding the mechanisms of interaction and regulation of eosinophils across different tissues, the specific molecular mechanisms remain incompletely elucidated. A deeper comprehension of the pathogenic roles of eosinophils in distinct spatiotemporal contexts will also aid in guiding future therapeutic strategies for eosinophilic disorders and certain systemic inflammatory diseases.

## 6 Discussion

As a key immune cell in mucosal immunity, gastrointestinal eosinophils have evolved from being perceived as mere terminal effector cells to being recognized as bidirectional regulators with plasticity, involved in both immune responses and tissue homeostasis maintenance. Understanding the diverse functions and subpopulations of eosinophils is crucial for elucidating the pathogenesis of eosinophilic diseases and identifying appropriate therapeutic targets. Currently, in addition to traditional therapies, novel biologics targeting eosinophil clearance (e.g., Siglec-8, IL-5, IL-4) and chimeric antigen receptor T-cell (CAR-T) therapy have entered the exploratory phase (140), potentially offering new treatment options for refractory and relapsed patients. Clinical trials have demonstrated that monoclonal antibodies targeting eosinophil clearance can effectively reduce eosinophil infiltration in peripheral blood and tissues, although there are significant individual variations in clinical symptom improvement. However, the efficacy and safety of CAR-T therapy in EoE remain to be established. These limitations underscore the importance of understanding the mechanisms underlying the diverse functions and subtypes of eosinophils, as well as their interactions with other cells and tissues during homeostasis and pathogenesis, to achieve precise treatment of gastrointestinal eosinophilic diseases.

## 7 Future perspectives

Despite recent advances in understanding eosinophil biology in the gastrointestinal tract, several critical knowledge gaps remain that require prioritization. First, molecular endotyping is essential to explain heterogeneous clinical trial responses. Transcriptomic profiling has revealed that eosinophilic gastritis (EoG) shares only 7% of its transcriptome with EoE, and eosinophilic colitis (EoC) exhibits minimal type 2 immune signatures, suggesting distinct pathogenic drivers that likely influence treatment responsiveness (141). Pre-treatment molecular stratification should therefore be incorporated into future biologic trials. Second, the microbiota has emerged as a critical determinant of intestinal eosinophil plasticity, capable of both promoting and restraining eosinophil responses in a niche- and context-dependent manner. Future investigations should prioritize deciphering how specific microbial species, strains, and their metabolites program eosinophil functional states across distinct intestinal regions and disease contexts (142). Deciphering these microbiota–eosinophil circuits may unlock novel microbiome-targeted interventions. Third, given the high clinical heterogeneity and rarity of non-esophageal Eosinophilic gastrointestinal disorders (EGIDs) (e.g., eosinophilic gastroenteritis and colitis), systematic understanding of region-specific eosinophil specialization, lifespan heterogeneity, and functional subsets across intestinal segments remains severely limited. Furthermore, existing knowledge largely derives from esophageal or animal studies, and large-scale, multicenter, multi-omic investigations in non-esophageal EGIDs are lacking, which hinders elucidation of their pathophysiological mechanisms and tissue-specific regulatory networks, underscoring an urgent need for translational research (141). Finally, monitoring solely by eosinophil counts fails to capture long-term tissue remodeling (fibrosis, smooth muscle dysfunction). Emerging non-invasive biomarkers—such as serum extracellular matrix turnover products (PRO-C3, C3M, C6M) and esophageal string test-derived EDN—show promise for assessing disease activity and may be adapted to track fibrotic progression (142, 143). Integrating these molecular and minimally invasive tools will enable precise, durable therapies across the full spectrum of EGIDs.
